# Infantile-onset Pompe disease complicated by sickle cell anemia: Case report and management considerations

**DOI:** 10.3389/fped.2022.944178

**Published:** 2022-09-28

**Authors:** Rodrigo Tzovenos Starosta, Ying-Chen Claire Hou, Katelyn Leestma, Prapti Singh, Luke Viehl, Linda Manwaring, Jorge Luis Granadillo, Molly C. Schroeder, Jamie N. Colombo, Halana Whitehead, Patricia Irene Dickson, Monica L. Hulbert, Hoanh Thi Nguyen

**Affiliations:** ^1^Division of Clinical Genetics and Genomics, Department of Pediatrics, Washington University School of Medicine, St. Louis, MO, United States; ^2^Department of Pathology and Immunology, Washington University School of Medicine, St. Louis, MO, United States; ^3^Division of Newborn Medicine, Department of Pediatrics, Washington University School of Medicine, St. Louis, MO, United States; ^4^Division of Pediatric Cardiology, Department of Pediatrics, Washington University School of Medicine, St. Louis, MO, United States; ^5^Division of Pediatric Hematology and Oncology, Department of Pediatrics, Washington University School of Medicine, St. Louis, MO, United States

**Keywords:** glycogen storage disorder type II, alpha-glucosidase, sickle cell anemia, immune tolerance induction, enzyme replacement therapy, methotrexate, newborn screening

## Abstract

Infantile-onset Pompe disease (IOPD) is a rare, severe disorder of lysosomal storage of glycogen that leads to progressive cardiac and skeletal myopathy. IOPD is a fatal disease in childhood unless treated with enzyme replacement therapy (ERT) from an early age. Sickle cell anemia (SCA) is a relatively common hemoglobinopathy caused by a specific variant in the hemoglobin beta-chain. Here we report a case of a male newborn of African ancestry diagnosed and treated for IOPD and SCA. Molecular testing confirmed two *GAA* variants, NM_000152.5: c.842G>C, p.(Arg281Pro) and NM_000152.5: c.2560C>T, p.(Arg854^*^) in *trans*, and homozygosity for the *HBB* variant causative of SCA, consistent with his diagnosis. An acute neonatal presentation of hypotonia and cardiomyopathy required ERT with alglucosidase alfa infusions preceded by immune tolerance induction (ITI), as well as chronic red blood cell transfusions and penicillin V potassium prophylaxis for treatment of IOPD and SCA. Clinical course was further complicated by multiple respiratory infections. We review the current guidelines and interventions taken to optimize his care and the pitfalls of those guidelines when treating patients with concomitant conditions. To the best of our knowledge, no other case reports of the concomitance of these two disorders was found. This report emphasizes the importance of newborn screening, early intervention, and treatment considerations for this complex patient presentation of IOPD and SCA.

## Introduction

Pompe disease (MIM #232300), also known as “glycogen storage disease type II”, is an autosomal recessive inborn error of glycogen metabolism that leads to lysosomal storage of undigested glycogen in muscle tissues. It is caused by a deficiency of acid alpha-glucosidase (GAA; EC 3.2.1.20), coded by *GAA* on chromosome 17q25.3. Pompe disease is generally categorized according to the age of onset of clinical manifestations: infantile-onset Pompe disease (IOPD) is characterized by onset of generalized hypotonia, macroglossia, and left ventricular hypertrophy before 12 months of age, and it generally leads to death by respiratory and cardiac failure before the second year of age unless early enzyme replacement therapy (ERT) is instituted (1, 2). Late-onset Pompe disease (LOPD), on the other hand, presents as proximal skeletal muscle weakness that progresses to involve bulbar, respiratory, and distal skeletal musculature as well as visceral smooth muscle, with relative sparing of cardiac muscle (3). Alglucosidase alfa, a recombinant form of acid alpha-glucosidase, is the only ERT modality currently available for the treatment of IOPD and has been shown to increase overall survival and ventilator-free survival, as well as to decrease left ventricular mass and increase left ventricular ejection fraction in patients with classic IOPD (4). The standard, FDA-approved alglucosidase alfa dose is 20 mg/kg every 2 weeks (4), although there is increasing evidence of improved outcomes with administration of higher doses (5–7). Before starting ERT for IOPD patients, the cross-reactive immunological material (CRIM), which is the presence of protein epitopes derived from acid alpha-glucosidase remnants in peripheral blood, needs to be ascertained as CRIM-negative individuals are at higher risk for developing sustained titers of anti-alglucosidase alpha neutralizing antibodies which can lead to treatment resistance and poorer outcomes (8). To prevent the development of anti-ERT antibodies, different immune tolerance induction protocols are used for CRIM-negative (9) and CRIM-positive (10) patients.

Sickle cell anemia (SCA, MIM #603903) is an autosomal recessive disorder of hemoglobin structure caused by the common *HBB* pathogenic variant (NM_000518.5):c.20A>T, p.(Glu7Val) which causes polymerization of deoxyhemoglobin, resulting in chronic intravascular hemolysis and intercurrent vaso-occlusive episodes. The chronic anemia of SCA requires cardiovascular adaptation with increased cardiac output to maintain tissue oxygen delivery, and individuals with SCA demonstrate increased stroke volume, left ventricle dilatation, and left ventricular hypertrophy starting in childhood and proportional to the severity of anemia (11, 12). Individuals with SCA also demonstrate decreased muscle microvascular oxygen delivery, decreased growth velocity, and lower muscle mass compared with healthy children (12, 13).

In this article, we report a patient diagnosed with IOPD and SCA. Although treated with ERT since the first month of life, his clinical course was complicated by left ventricular hypertrophy followed by early-onset dilation in infancy. This is a unique case where both conditions co-occurred in a patient with a severe clinical presentation and course.

## Case report

**Figure 1:**
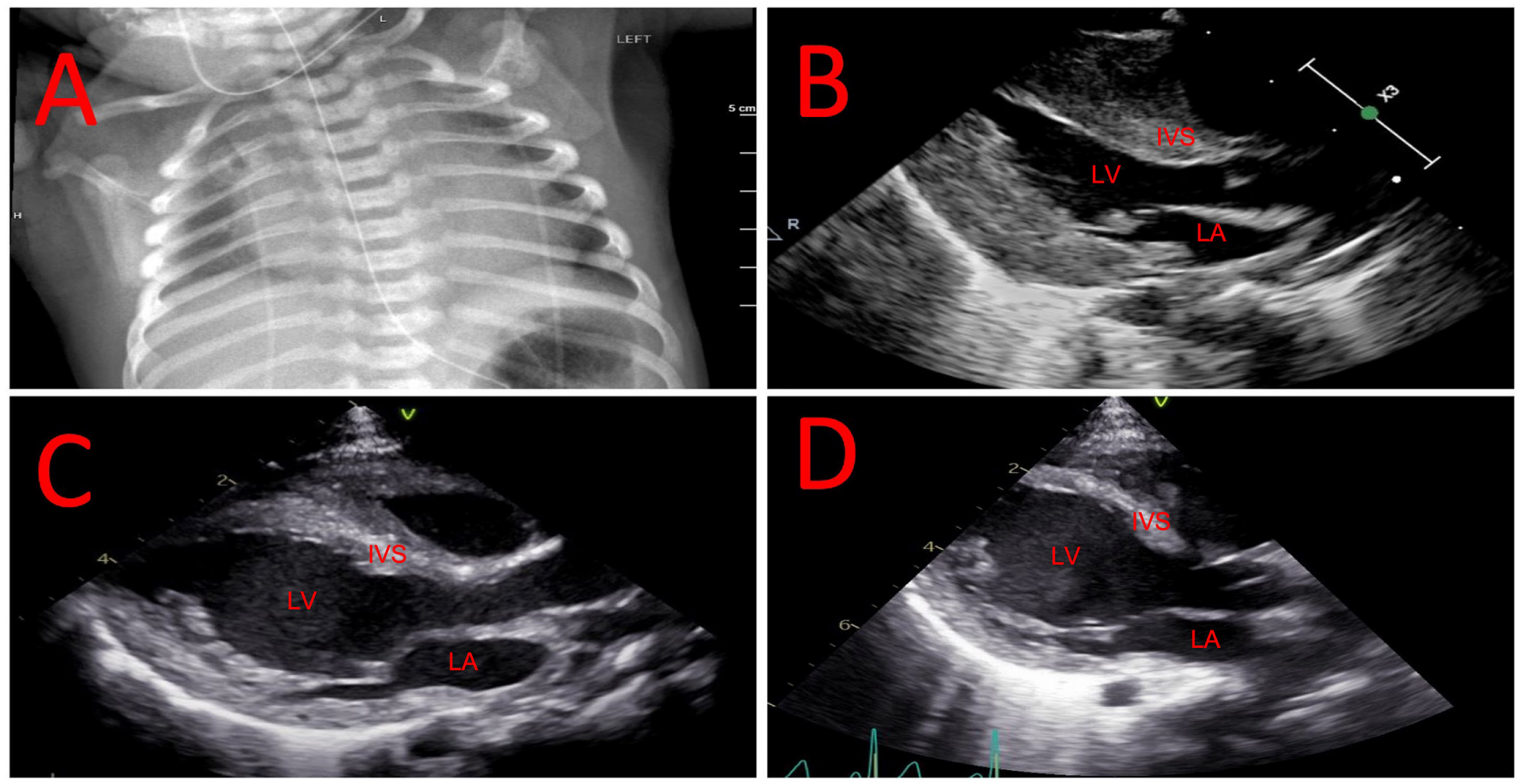
Evolution of cardiac hypertrophy and dilation. **(A)** Chest radiograph showing marked cardiomegaly in the neonatal period. **(B)** Echocardiogram showing hypertrophic left ventricle in the neonatal period. **(C)** Echocardiogram showing mixed left ventricular hypertrophy and dilation at 2 months of age. **(D)** Echocardiogram showing resolution of left ventricular hypertrophy with worsening of dilation at 7 months of age. IVS, interventricular septum; LA, left atrium; LV, left ventricle.

A male newborn of African American ethnicity was born at 39 weeks gestational age from non-consanguineous parents, both previously diagnosed as heterozygous carriers for the *HBB* c.20A>T, p.(Glu7Val) variant. The pregnancy was otherwise uncomplicated. Shortly after birth, he developed respiratory distress requiring transient non-invasive respiratory support and admission to the neonatal intensive care unit. A chest radiograph exhibited marked cardiomegaly (Figure 1A) and physical exam was significant for hypotonia and mild macroglossia. An echocardiogram showed severe left ventricular hypertrophy (Figure 1B). Further studies revealed elevated pro B-type natriuretic peptide (pro-BNP) at 25,701 pg/mL (normal <10,000 pg/mL), creatine-kinase (CK) at 1,374 U/L (normal <300 U/L), and aldolase at 37.8 U/L (normal 0.1−8 U/L). Work-up for other metabolic and genetic diseases, including acylcarnitine profile, total and free carnitine, ammonia, lactate, serum amino acids, and urine organic acids, was non-contributory. Karyotype and chromosomal microarray did not detect abnormalities. A cardiomyopathy next-generation sequencing (NGS) panel analyzing the coding regions of 163 genes associated with cardiac disease was performed at the Washington University Genomic and Pathology Services. Two *GAA* variants, NM_000152.5: c.842G>C, p.(Arg281Pro) and NM_000152.5: c.2560C>T, p.(Arg854^*^) were found in compound heterozygosity. While the genetic panel was pending, newborn screening (NBS) showed a decreased GAA enzyme activity at 4% (normal >22%). The hemoglobinopathy portion of the NBS showed absence of Hb A and presence of Hb F and S, consistent with SCA. Confirmatory GAA enzyme activity was low at 0.8 pmol/punch/h (normal >3.88) and urine glucotetrasaccharide (Hex4) was elevated at 29.2 mmol/mol creatinine (normal <20) on day of life (DOL) 14. CRIM status determined by Western blotting was positive. The patient was started on alglucosidase alfa infusions (20 mg/kg every 2 weeks) with an immune tolerance induction protocol comprised of methotrexate at 0.4 mg/kg for three consecutive days, starting on the day prior to the infusion, on DOL 30. This protocol was used for the first three enzyme replacement therapy (ERT) infusions (total of 9 doses of methotrexate), as previously described (10).

The SCA diagnosis was confirmed by *HBB* sequencing showing homozygosity for c.20A>T and negative *HBB* duplication/deletion analysis, ruling out hereditary persistence of fetal hemoglobin. He was started on penicillin V potassium prophylaxis at 1 month old. Due to concern that chronic anemia would adversely impact his cardiac function, and that any SCA vaso-occlusive complications such as acute chest syndrome or splenic sequestration in infancy would be poorly tolerated, chronic red blood cell transfusion therapy was chosen as his primary disease-modifying therapy. His first transfusion was administered at 2 months of age when Hb decreased to 8.4 g/dl. His transfusion therapy goal is to maintain Hb >9.5 g/dl to minimize the physiological stress of anemia. At 2 months of life, the patient was noticed to have new-onset left ventricular dilation in addition to cardiac hypertrophy (Figure 1C). Due to concerns for a decrease in left ventricular systolic function the patient was started on enalapril to prevent cardiac remodeling.

**Figure 2:**
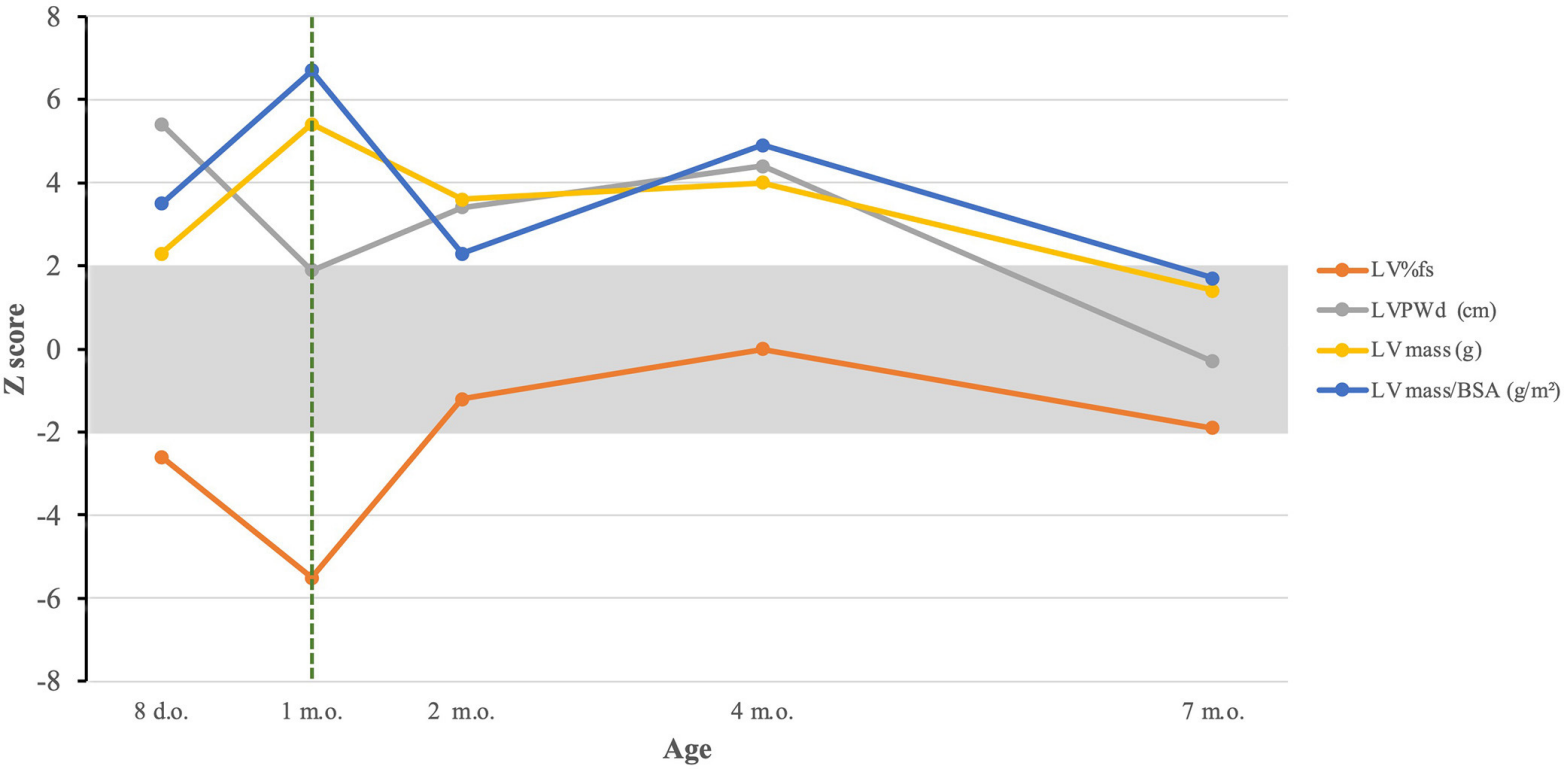
Evolution of cardiac indices in response to treatment. The dashed green line represents initiation of ERT. LV%fs, left ventricular percentual fractional shortening; LVPWd, left ventricular posterior wall dimension; LV mass, left ventricular mass; LV mass/BSA, left ventricular mass/body surface area.

He has had 3 readmissions for respiratory decompensation due to viral infections: dual SARS-CoV-2 and respiratory syncytial virus infection at 3 months old (requiring high-flow nasal cannula), human metapneumovirus infection at 5 months old (requiring high-flow nasal cannula in the ICU setting) and rhino/enterovirus at 6 months old (requiring BiPAP in the ICU setting). At 7 months old, his outpatient ERT dosing was noted to having been based solely on birth weight, which resulted in a progressively decreasing ERT dose per kg body weight; on his most recently outpatient infusions this was ~13 mg/kg, and was corrected to 20 mg/kg. He was admitted subsequently at 7 months old due to respiratory failure with concern for an aspiration event requiring admission to the intensive care unit for non-invasive respiratory support. A repeat echocardiogram showed resolution of left ventricular hypertrophy with continuing dilation (Figure 1D). Evolution of systolic function and cardiac indices are presented in Figure 2. He has received a lifetime total of 10 pRBC transfusions at the time of this report, with a hemoglobin nadir of 7.7 g/dL. He remains gastrostomy-dependent. He continues to exhibit very low axial and appendicular muscle tone. He has required ~150–160 kcal/kg/day of enteral nutrition since birth for growth; non-etheless, he was at <1% (Z-score = −2.53, WHO Boys 0–2 years) for weight and <1% (Z-score = −3.57, WHO Boys 0–2 years) for weight-for-length at his most recent admission.

## Discussion

IOPD, with early onset and a lethal prognosis, can be modified through intervention and therefore meets generally accepted consensus criteria for inclusion in NBS programs (14). In the United States of America, the inclusion of Pompe disease in NBS was recommended in 2015, and currently more than 20 states have started the necessary implementation steps (15). The incidence of Pompe disease is historically reported as ~1:40,000 live births Western countries (16); however, this epidemiology has been calculated mainly based on European populations, with blind studies showing a higher incidence in other populations including people of African ancestry (17). Moreover, after implementation of universal screening through NBS there have been many reports of an incidence up to 4x higher than expected [as summarized by Davids et al. (18)], pointing to a substantial amount of historically missed diagnoses. SCA, also an autosomal recessive disorder included in NBS, contrasts with Pompe disease by having a high populational prevalence, with ~100,000 diagnosed Americans, of whom ~90% are of African ancestry (19). Prenatal genetic diagnosis for SCA and IOPD, as for most genetic disorders, is still incipient but may allow for even swifter initiation of treatment in future patients. In this article, we have presented a case of concomitant Pompe disease—presenting as IOPD—and SCA. The lack of previous reports of this co-incidence may reflect an epidemiological gap and probable underdiagnosis of Pompe disease in people of African ancestry, reinforcing previous studies (17) and underscoring the importance of universal NBS for equanimity in healthcare.

Two heterozygous *GAA* variants were detected. The p.(Arg854^*^) variant is predicted to result in a premature stop codon, nonsense-mediated decay, and has been demonstrated to result in an absent gene product (20); this variant has been observed in homozygous or compound heterozygous states in CRIM-negative individuals diagnosed with Pompe disease (21, 22). Published functional studies of the p.(Arg845^*^) variant have shown negligible activity compared to wild-type protein (23). This variant is commonly observed in individuals of African descent with IOPD (24, 25). This variant meets criteria PVS1, PM2, PM3, and PP4 of the American College of Medical Genetics/Association for Molecular Pathology (ACMG/AMP) guidelines for the interpretation of sequence variants (26) and is thus classified as pathogenic. We also identified a novel variant, c.842G>C, p.(Arg281Pro), in this patient, not previously observed in individuals with Pompe disease. A different amino acid change at the same codon, c.841C>T, p.(Arg281Trp), has been reported as a known likely pathogenic variant (27). Our patient's variant is absent in gnomAD, and multiple *in silico* prediction algorithms are in agreement that this variant may have a deleterious/probably damaging effect on protein function. Given the available evidence and in accordance with the ACMG/AMP guidelines, as well as the ClinGen *GAA* sequence analysis recommendations from ClinGen, the *GAA* p.(Arg281Pro) variant meets criteria PM2, PM3, PM5, PP3, and PP4 and was classified as likely pathogenic. Segregation studies on parental sample further confirmed that the p.(Arg845^*^) and p.(Arg281Pro) are in *trans* configuration, consistent with biallelic disruption of the gene product.

Treatment of IOPD with alglucosidase alfa ERT has been shown to improve survival, functionality, cardiomyopathy, and weight gain of individuals with IOPD (28), with the better outcomes observed in patients started on ERT in the first month of life (29), as was the case in our patient. In contrast with most other lysosomal diseases, the start of treatment in IOPD is contingent upon determination of CRIM status for appropriate immune tolerance induction as without immunomodulation, virtually all CRIM-negative patients develop a high sustained immune response to ERT that leads to dismal outcomes (9, 30). Although approximately half of CRIM-positive patients do not have significant antibody titers, 32–40% of CRIM-positive patients who receive ERT without immune tolerance induction can develop intermediate- or high sustained antibody titers, leading to worse outcomes when compared to CRIM-positive patients that received immunomodulating agents (8, 31). Currently, the validated immune tolerance induction regimen for CRIM-positive patients contains methotrexate (10), a potent myelotoxic folate antagonist that can lead to myelosuppression. Although this regimen utilizes low-dose, short-term methotrexate, which is generally welltolerated in IOPD patients, this may not be the most suitable regimen for a patient with concomitant bone marrow disease. Evidence for the use of methotrexate in patients with SCA is scarce and includes reports of transient cytopenia despite the concomitant administration of folinic acid (32). In this case report, anemia—which may have been exacerbated by the use of methotrexate—may have led to worsening cardiac dilatation despite ERT, highlighting the complexity of medical care in individuals with a dual diagnosis. As the incidence of Pompe disease increases as a result of universal NBS, more patients with concurrent IOPD and SCA (or other hemoglobinopathies) are expected to be diagnosed, and it becomes necessary to study novel immune tolerance induction protocols without the use of methotrexate, or with maximized support (with agents such as leucovorin or with preventive blood transfusions) for safe ERT initiation in this population. As newer recombinant enzymes and gene therapies are being tested, it will be important that ITI regimens are also validated for co-morbid populations.

Finally, decisions about SCA management were made based on knowledge that growth and cardiac function are more normal in children with SCA who have higher hemoglobin concentrations. Typically, chronic red blood cell transfusion therapy is utilized in SCA for stroke prevention with a primary goal of maintaining Hb S <30%, which may result in a nadir Hb pre-transfusion of <8 g/dl (33). In the current case, since this degree of anemia may compromise growth and increase the risk of cardiac stress, a transfusion strategy similar to that utilized in children with beta-thalassemia major was chosen, in which total hemoglobin concentration is maintained at 9–10 g/dl to minimize anemia-related organ dysfunction (34). Chronic transfusions will cause iron overload, which over many years can increase risk of heart failure but is manageable with iron chelating drugs (34). Hydroxyurea is another option for disease-modifying therapy, but it is not recommended until age 9 months. Patients with SCA have varying response to hydroxyurea, but in general the Hb remains higher on chronic red blood cell transfusion therapy than on this medication (33). Converting to hydroxyurea therapy from transfusion therapy may be a possibility if cardiac function remains stable and his strength is improving as he gets older, with careful monitoring of cardiac and skeletal muscle function. Hematopoietic stem cell transplantation can be curative for sickle cell disease; however, it has a delicate risk/benefit balance and requires a good functional status, thus not being a feasible option for this patient.

## Conclusion

We have presented a unique case of concomitant IOPD and SCA leading to complex initiation of ERT and need for aggressive transfusion support to prevent cardiomyopathy due to anemia. As more locations implement NBS for Pompe disease, it is expected that such cases will become more common, and novel protocols that take the myelotoxicity of immune tolerance induction regimens into account will become necessary.

## Data availability statement

The original contributions presented in the study are included in the article/supplementary materials, further inquiries can be directed to the corresponding author.

## Ethics statement

Ethical review and approval was not required for the study on human participants in accordance with the local legislation and institutional requirements. Written informed consent to participate in this study was provided by the participants' legal guardian/next of kin.

## Author contributions

RS wrote the first version of the manuscript. RS, PS, JG, and PD saw the patient at presentation, made the clinical and biochemical diagnosis of Pompe disease and started immune tolerance induction, and enzyme replacement therapy. RS, KL, and HN continued to follow the patient at the genetic clinic appointments, infusions, and admissions. LM saw the patient at follow-up and counseled the family. Y-CH and MS made the molecular diagnosis and wrote the variant analysis. MH made the diagnosis of sickle cell anemia, the decision for chronic transfusion therapy, and has been following the patient throughout life. JC made all the cardiological diagnoses and has been following the patient throughout life. LV and HW have been following the patient from a critical care perspective throughout life. All other authors provided feedback on the manuscript, reviewed it, and approved the final version and the decision to submit it for publication.

## Funding

Funds for publication were provided to PD by Washington University in Saint Louis.

## Conflict of interest

Author PD declares the following conflicts of interest: Genzyme: research support, M6P Therapeutics: research support, and Mandos Health: consulting. Author MH declares the following conflict of interest: Pfizer, Inc: spouse employment, Bluebird Bio: consulting, Forma Therapeutics: research funding, Global Blood Therapeutics: research funding and consulting. The remaining authors declare that the research was conducted in the absence of any commercial or financial relationships that could be construed as a potential conflict of interest.

## Publisher's note

All claims expressed in this article are solely those of the authors and do not necessarily represent those of their affiliated organizations, or those of the publisher, the editors and the reviewers. Any product that may be evaluated in this article, or claim that may be made by its manufacturer, is not guaranteed or endorsed by the publisher.
